# Mitochondrial DNA Variability of Domestic River Buffalo (*Bubalus bubalis*) Populations: Genetic Evidence for Domestication of River Buffalo in Indian Subcontinent

**DOI:** 10.1093/gbe/evv067

**Published:** 2015-04-20

**Authors:** Muniyandi Nagarajan, Koodali Nimisha, Satish Kumar

**Affiliations:** ^1^Department of Genomic Science, School of Biological Sciences, Central University of Kerala, Kasaragod, Kerala, India; ^2^CSIR-Centre for Cellular and Molecular Biology, Hyderabad, Telangana, India

**Keywords:** mtDNA, d-loop, buffalo, livestock, phylogeography, genetic diversity

## Abstract

River buffalo, *Bubalus bubalis* is a large bovine species frequently used livestock in southern Asia. It is believed that the river buffalo was domesticated from *Bubalus arnee*, the wild buffalo of mainland Asia, a few thousand years ago, probably during the period of Indus Valley civilization. However, the domestication history of the river buffalo has been the subject of debate for many decades mainly due to the lack of clear archeological evidence and the divisive conclusions of the genetic studies. Therefore, in order to understand the domestication history and genetic relationship among the various river buffalo populations, we analyzed 492-bp region of mitochondrial DNA control region sequences of 414 river buffalo sampled from India, Pakistan, Egypt, and Iran along with the available 403 swamp buffalo sequences. The phylogenetic analyses of our study along with the archaeological evidence suggest that the river buffalo was domesticated in an atypical manner involving continuous introgression of wild animals to the domestic stocks in Indian subcontinent prior to mature phase of Indus Valley civilization (2600–1900 BC). Specifically, our data exclude Mesopotamian region as the place of domestication of the river buffalo.

## Introduction

Water buffalo, *Bubalus bubalis* is one of the most important livestock species in several Asian countries and is used for milk, meat, and agricultural purposes. Based upon morphological, ecological, behavioral, cytological, and molecular genetics attributes buffalo are classified into two types, namely, river and swamp buffalo (Cockrill 1981; Kumar, Nagarajan, Sandhu, Kumar, Behl, and Nishanth 2007). The former is widely distributed in the Indian subcontinent, Middle-east, Eastern Europe and North Africa, whereas the latter is found in the Northeast India, Bangladesh, China and Southeast Asian countries (Cockrill 1981). The river buffalo in India (98 million), Pakistan (26 million), and Egypt (3.9 million) constitutes about 90% of the total global buffalo population and accounts for 92% of the total milk produced from this species (FAOSTAT 2006).

Recent molecular genetic markers studies using mitochondrial DNA (mtDNA) and the present-day distribution of these two types have settled that swamp and river buffalo have been domesticated independently (Kumar, Nagarajan, Sandhu, Kumar, Behl, and Nishanth 2007; Yindee et al. 2010). Generally, it is believed that both river and swamp domestic buffalo were derived from *Bubalus arnee.* Based upon mitochondrial control region and cytochrome *b* sequence analysis we have earlier proposed that these two domestic types would have been derived independently from their respective wild ancestors that would have differed from each other at least at the level of subspecies (Kumar, Nagarajan, Sandhu, Kumar, Behl, and Nishanth 2007). Seals dating around 2500 BC from Harappan culture and similar age seals from Akkadian era (2100–2500 BC) of Mesopotamia civilization have indicated that buffalo would have been domesticated at least prior to this period (Boehmer 1975). Cockrill (1981) suggested that river buffalo was domesticated for the first time in Indus Valley and Mesopotamia civilizations between 2000 BC and 3000 BC. Toward the end of Akkadian period buffalo disappeared from Mesopotamia iconography and was seen again after a gap of nearly 2000 years during Sasanian era (Potts 1997). Boehmer (1975) has suggested that original introduction of domestic buffalo would have been a gift from Indus Valley civilization to Mesopotamia. There is no clear archeological evidence whether wild buffalo was present and domesticated in Mesopotamia or wild buffalo was first domesticated in Indus Valley civilization and then subsequently was transported from there. The buffalo remains have been recovered from Kutch-Dholavira, dating back to mid third millennium BC, which have been believed to be domestic buffalo because of the size differences observed in the Dholavira and Santhli/Mehargarh buffalo remains (Patel and Meadow 1998). Depiction of buffalo figures in both Mesopotamia and Indus Valley iconography shows animals with crescent type horn and at times this morphological feature is taken as implicit evidence that these animals would have been of swamp type. This is unlikely to be true as Toda buffalo from Nilgiri hills in South India are riverine type (Nair et al. 1986; Kumar, Nagarajan, Sandhu, Kumar, and Behl 2007) but have horn type similar to those depicted in the iconography of both Indus Valley and Mesopotamia civilizations. Our recent studies on mitochondrial control region indicate that if Indian subcontinent region was the place of domestication of river buffalo, the Northwestern region of India was the most likely place for this to happen (Kumar, Nagarajan, Sandhu, Kumar, and Behl 2007). To understand the domestication history of river buffalo further, we have now analyzed 492-bp mtDNA control region sequences of 414 domestic river buffalo sampled from India, Pakistan, Egypt, and Iran along with the available 403 swamp sequences and provide genetic evidence that river buffalo was domesticated in Indian subcontinent and domestic river buffalo would have reached Mesopotamia in ancient times from India.

## Results and Discussion

**Fig. 1.—:**
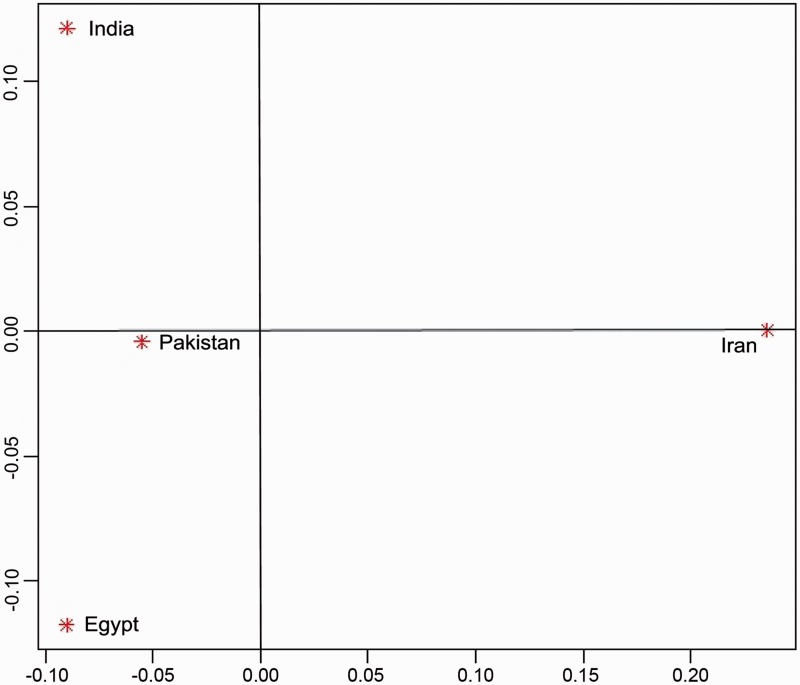
MDS plot. The MDS plot was drawn using pairwise *F*_ST_ values, which shows the overall genetic relationship of four domestic river buffalo populations based on 492-bp mtDNA control region sequences.

This study provides the first comprehensive report for domestication history of river buffalo using mtDNA control region sequences. A total of 151 unique haplotypes were found from 74 variable sites in 414 buffalo sequences from four buffalo populations. The haplotype diversity ranged from 0.8236 ± 0.0488 in Egyptian population to 0.9428 ± 0.0088 in Indian population (table 1). First, analysis of molecular variance (AMOVA) test was performed based on geographical distribution of the buffalo populations, which showed 91% variation within the population and only 9% variation between populations. Further AMOVA test was performed for several combinations among four river buffalo populations and the results remained same or not significant except in one combination where the Indian, Pakistani, and Egyptian buffalo were considered as a single group against Iranian buffalo. Notably 30% variation was observed between these two groups, which indicated that Iranian buffalo is significantly different from the remaining populations. Among the four populations, the highest *F*_ST_ value was obtained between Egyptian and Iranian buffalo populations whereas the lowest *F*_ST_ value was obtained between Pakistani and Egyptian buffalo populations. Further, the pairwise *F*_ST_ values were analyzed using multidimensional scaling (MDS) plot, in which the Indian, Pakistani, and Egyptian buffalo populations closely resided at one place of the plot and the Iranian buffalo stood alone on the other side of the plot which strongly support AMOVA results (fig. 1).

**Table 1:** Geographic Distribution and Haplotype Diversity of River Buffalo

S. No	Population	Number of Samples	Number of Haplotypes	Diversity (SE)
1	India	217	82	0.9428 ± 0.0088
2	Pakistan	123	49	0.9126 ± 0.0162
3	Egypt	48	16	0.8236 ± 0.0488
4	Iran	26	17	0.9385 ± 0.0339

**Fig. 2.—:**
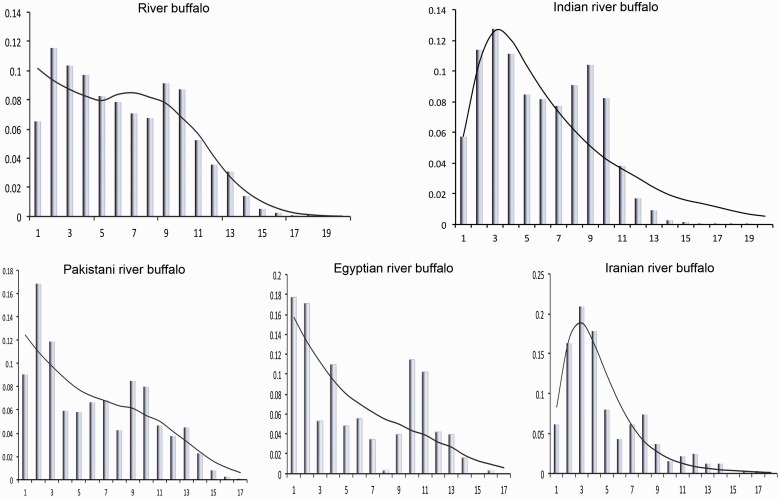
Mismatch distributions of mtDNA control region sequences (492 bp) of domestic river buffalo. The observed mismatch distributions (bars) are compared with the expected mismatch distributions (solid lines) under sudden expansion model. The number of nucleotide differences between a pair of sequences is indicated along the *x* axis, and the respective frequency (%) is shown along the *y* axis.

In domestic animals, it is expected that after domestication event the founding haplotypes would expand in numbers to give rise to a population and this would be reflected in the mismatch distribution curve (Rogers and Harpending 1992). When four buffalo populations were considered as a single population, the mismatch distribution curve was bimodal which suggests strongly population subdivision (fig. 2). Mismatch distribution curve was also obtained for each population separately. Interestingly, the mismatch distribution curve obtained based on the geographical distribution showed a mixed pattern. The Indian and Iranian buffalo populations showed unimodal distribution curve with a peak around at three differences and raggedness value 0.007 and 0.027, respectively, suggesting expansion of domestic buffalo population in India and Iran (fig. 2). A highly significant negative value of Fu’s Fs statistics (−25.19 and −8.91) also further supported the demographic expansion. The Pakistani and Egyptian buffalo showed bimodal mismatch distribution, which indicates fragmentation in Pakistani and Egyptian buffalo populations (fig. 2).

**Fig. 3.—:**
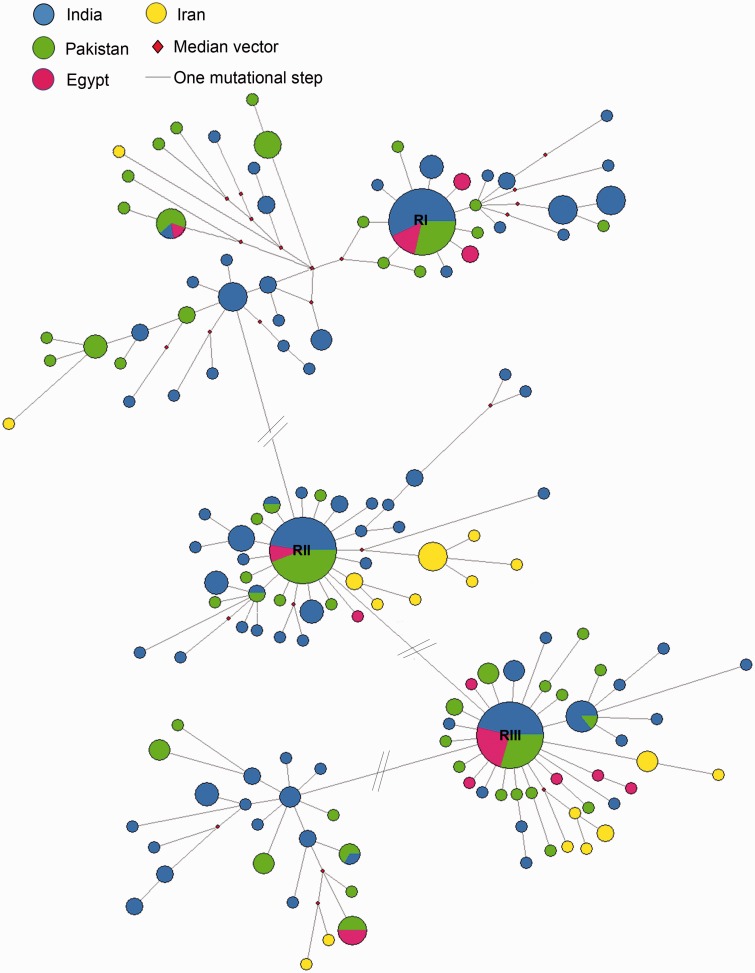
Reduced median network of domestic river buffalo based on 492-bp control region sequence. Each haplotype is represented by a circle and the area of the circle is proportional to its frequency. Samples from different regions are mentioned in different colors. The length of each branch is proportional to the number of mutations on the respective branch. The # indicates that there is only one mutational step between the two haplotypes, the line has been broken to the convenient arrangement of the each nodes.

The maximum parsimony (MP) and Bayesian trees were constructed using 151 unique river buffalo haplotypes and a *Bos taurus* sequence was used to root the phylogenetic tree. All the 151 haplotypes formed a single major clade and within the major clade a few minor clades were observed in the MP and Bayesian trees (supplementary figs. S1 and S2, Supplementary Material online). The reduced median network was constructed using 414 river buffalo sequences (fig. 3). The network was complex and revealed at least three major expanding haplotypes RI (present in 35 buffalo), RII (present in 59 buffalo), and RIII (present in 78 buffalo) with star-like appearances. Among the three major expanding haplotypes two of them (RII and RIII) were interconnected to each other with one mutational step. The RI haplotype was connected with the main network through a median vector and differed at least six mutational steps from the haplotypes RII and RIII. Indian, Pakistani, and Egyptian buffalo shared the haplotypes extensively with each other and showed their presence in all the three major expanding haplotypes. On the other hand, the Iranian buffalo did not share the haplotype with Indian, Pakistani, and Egyptian buffalo and mostly appeared as a singleton around the RII and RIII haplotypes. However, the average sequence divergence was only 0.4% between RIII haplotype (major haplotype) and Iranian buffalo whereas the average sequence divergence between RI haplotype and Iranian buffalo was 1.4%.

The Iranian buffalo was mostly found around RII and RIII haplotypes and not found in and around RI haplotype, which suggest that 1) the migration of river buffalo from India to Iran is ancient followed by genetic drift would have played vital role for the high genetic variability in Iranian buffalo and 2) there was a continual influx of wild buffalo into the Indian domestic stocks after the initial domestication event, which was absent in Iranian buffalo, which is another possible reason for the high genetic variability between the Indian and Iranian buffalo populations. To obtain more insight into river buffalo domestication, we constructed the network for each population separately and two maternal lineages were observed in Indian, Pakistani, and Egyptian buffalo populations whereas this dichotomy was not observed in Iranian buffalo (data not shown). Further, the star-like expansion was also not observed in the reduced median network of Iranian buffalo and mostly appeared as singleton. The lack of haplotype sharing of Iranian buffalo with other buffalo populations, especially with Pakistani buffalo, suggests that the migration of river buffalo from India to Iran could have not occurred through land but probably through sea as elephant (Bertman 2003).

The buffalo seals obtained in Indus Valley were dating back to 3000 BC or even earlier, which indicates that the buffalo had already been domesticated in the Indian subcontinent around that time (Zeuner 1963; Cockrill 1981). But, the river buffalo has been noticed in Iran during 2500 BC and then it has become one of the most important domestic animals in Iran (Naserian and Saremi 2007). Similarly, buffalo was unknown to Egypt during the time of Pharaohs. The Arabs and pilgrimis probably introduced it to Egypt after ninth century (Sidki 1951) and the occurrence of RI, RII, and RIII haplotypes in Egyptian buffalo provides support for the recent migration of buffalo to Egypt. Further, the low *F*_ST_ (0.0087) and AMOVA (0.87%) values between Pakistani and Egyptian buffalo suggest that buffalo from Pakistan region could have been used to spread buffalo in Egypt. It has been established that the genetic variability of the domesticated species would be high at the place of its origin. Among the four buffalo populations studied, Indian buffalo showed high level of genetic variability, which suggests that the domestication occurred in the present-day Indian region. Within the Indian breeds, the haplotype diversity was high for the Mehsana (0.9829 ± 0.0139), Surati (0.9581 ± 0.0208), and Pandharpuri (0.9829 ± 0.0154) breeds sampled from Northwestern parts of India (place of Indus Valley civilization). Therefore, the present analysis of Indian samples along with those from other countries strongly suggests that the river buffalo was domesticated in Indian subcontinent, particularly in the Northwestern region of India and the places of distribution of the present-day Mehsana, Surati, and Pandharpuri breeds would have been the most likely candidate.

**Fig. 4.—:**
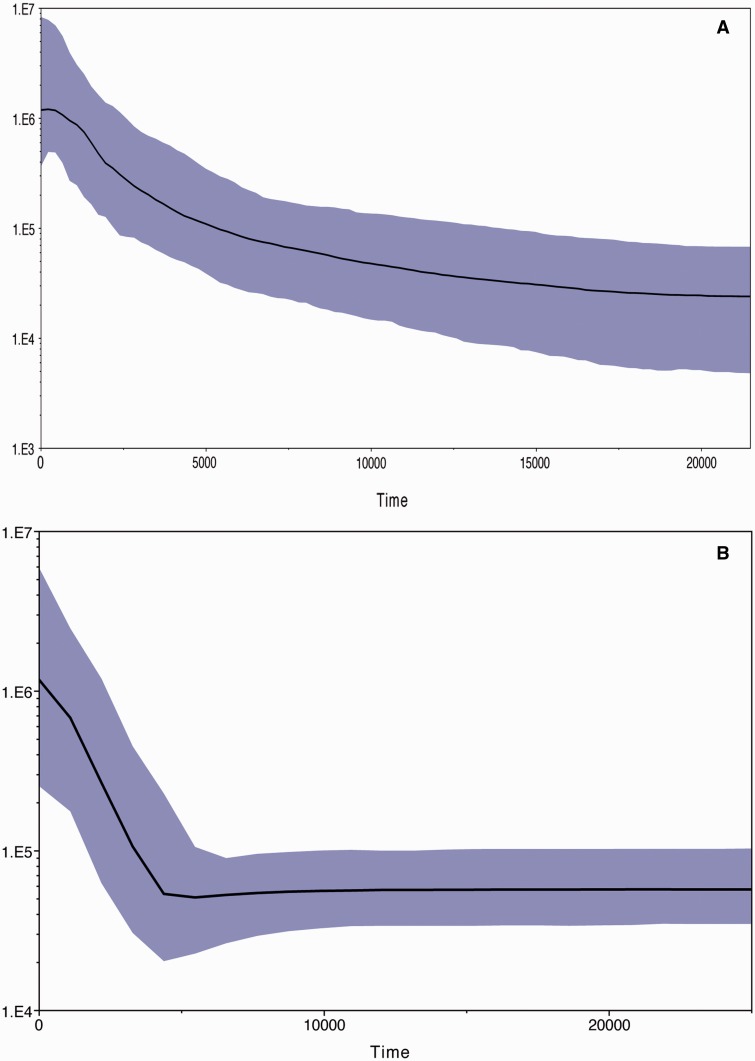
BSP comparing the river and swamp buffalo population size through time based using mtDNA control region sequences. (*A*) BSP of river buffalo. (*B*) BSP of swamp buffalo. The solid black lines are the median estimate and shaded areas (pink) represent the 95% upper and lower highest posterior density intervals. The *y* axis represents the female effective population size and the *x* axis represents time in years.

Although, there has been a “difference of opinion” over the time of buffalo domestication, a good number of studies have reported based on the archeological evidences that the buffalo domestication took place most likely in the Indus Valley civilization during third millennium BC (Zeuner 1963; Cockrill 1981; Gouin 1990, 1992). In order to find out the population expansion time of river and swamp buffalo, Bayesian skyline plot (BSP) was employed. BSP provides graphical representation of changes in population size over time. The BSP of swamp buffalo showed a constant population size until approximately 4,500 years BP followed by a rapid expansion until the present, whereas the BSP of river buffalo showed a constant population size until approximately 10,000 years BP followed by a steady increase in population size until approximately 2500 BP, and there after upward acceleration in population size until present (fig. 4). The lack of clear point of inflection in BSP of river buffalo supports our earlier conclusions that river buffalo has been domesticated in an atypical manner involving continuous introgression of wild animals in the domestic stocks over a long time unlike swamp buffalo. The BSP analysis provides the support to the view of Patel and Meadow (1998) that buffalo would have been domesticated prior to mature phase of Indus Valley civilization (2600–1900 BC). However, the place of domestication of swamp buffalo remains debated.

## Conclusions

Based on our present and earlier analyses and in the light of the available archeological evidence we propose that the river buffalo was domesticated in an atypical manner involving continuous introgression of wild animals to the domestic stocks in Indian subcontinent prior to mature phase of Indus Valley civilization, and the river buffalo was first domesticated in the Northwestern region of India from where it spread to other parts of the world. More notably our analysis excludes the Mesopotamian region as the place of domestication of the river buffalo. The BSP analysis further reconfirms our earlier findings that river and swamp buffalo were domesticated independently.

## Materials and Methods

We have analyzed 414 river buffalo mtDNA control region sequences representing India, Pakistan, Iran, and Egypt (table 1) to unravel the domestication history of the river buffalo. These 414 sequences were retrieved from the Gene Bank (supplementary table S1, Supplementary Material online). The complete mtDNA control region sequence was available for 291 river buffalo sampled from India, Iran, and Egypt, whereas only 505-bp mtDNA control region sequence was available for the Pakistani buffalo. Therefore, all the sequences were truncated into 492 bp size, which accommodated the hypervariable region I of the control region. The sequences were edited and aligned using AUTOASSEMBLER (Perkin Elmer) and ClustalX (Thompson et al. 1997) programs. The population genetic parameters, haplotype diversity, *F*_ST_ values, mismatch distribution, Fu’s Fs statistics, and AMOVA were estimated using ARLEQUIN (Schneider et al. 2002). The MDS plot was drawn for the pairwise *F*_ST_ values using package PSYCH in R statistical package (www.r-project.org). Phylogenetic trees were constructed based on 151 unique haplotypes of the river buffalo and a *Bos taurus* sequence (NC_006853) was used as an outgroup. Bayesian phylogenetic tree was constructed by MrBayes (Ronquist and Huelsenbeck 2003) using the general time reversible model with invariant site plus eight gamma categories. The Markov chain Monte Carlo (MCMC) chains were run for 10 × 10^6^ cycles. A total of 20,000 trees were sampled, and a 50% majority rule consensus tree was generated with burnin = 5,000. The tree construction was repeated three times. The MP tree was generated using the software MEGA (Kumar et al. 2004). The close-neighbor-interchange algorithm was employed with 1,000 bootstrapping values. The reduced median network was drawn for the 414 control region sequences using NETWORK program (Bandelt et al. 1995). The BSP method implemented in BEAST (Drummond et al. 2012) was used to infer the demographic history of river and swamp buffalo. We used a data set of 414 (492 bp) and 403 (506 bp) mtDNA control region sequences of river and swamp buffalo, respectively (supplementary table S1, Supplementary Material online) for BSP. The MCMC was run for 5 × 10^7^ iterations with a burn-in of 5 × 10^6^ under the Hasegawa–Kishino–Yano model with the substitution rate of 32% per nucleotide Myr^−^^1^ (Shapiro et al. 2004). The genealogies and model parameters were sampled every 5,000 iterations. All the operators were kept at default settings. The MCMC runs were repeated to refine skyline parameters and the convergence of the chains to the stationary distribution was confirmed by effective sample size (>100) for all parameters using TRACER (Rambaut et al. 2014).

## Supplementary Material

Supplementary figures S1 and S2 and table S1 are available at *Genome Biology and Evolution* online (http://www.gbe.oxfordjournals.org/).

Supplementary Data
