# Supplementary material for: Mitochondrial DNA Variability of Domestic River Buffalo (Bubalus bubalis) Populations: Genetic Evidence for Domestication of River Buffalo in Indian Subcontinent
Source: Genome Biol Evol. 2015 Apr 20;7(5):1252–9. doi: 10.1093/gbe/evv067 (PMC4453062; doi:10.1093/gbe/evv067)
Supplement: Supplementary Data [file supp_7_5_1252__index.html]

Mitochondrial DNA Variability of Domestic River Buffalo (Bubalus bubalis) Populations: Genetic Evidence for Domestication of River Buffalo in Indian Subcontinent — Mitochondrial DNA Variability of Domestic River Buffalo (Bubalus bubalis) Populations: Genetic Evidence for Domestication of River Buffalo in Indian Subcontinent — Supplementary Data 

# Mitochondrial DNA Variability of Domestic River Buffalo (*Bubalus bubalis*) Populations: Genetic Evidence for Domestication of River Buffalo in Indian Subcontinent

## Supplementary Data

files

**Files in this Data Supplement:**

- Supplementary Data - pdf file
- Supplementary Data - pdf file
- Supplementary Data - docx file
- Supplementary Data - docx file
