# Supplementary material for: Mitochondrial DNA Variability of Domestic River Buffalo (Bubalus bubalis) Populations: Genetic Evidence for Domestication of River Buffalo in Indian Subcontinent
Source: Genome Biol Evol. 2015 Apr 20;7(5):1252–9. doi: 10.1093/gbe/evv067 (PMC4453062; doi:10.1093/gbe/evv067)
Supplement: Supplementary Data [file supp_evv067_Figure_S1a.pdf]

Figure S1. Maximum parsimony phylogenetic tree of river buffalo rooted with *Bos taurus*. The bootstrap values are shown above the branches.

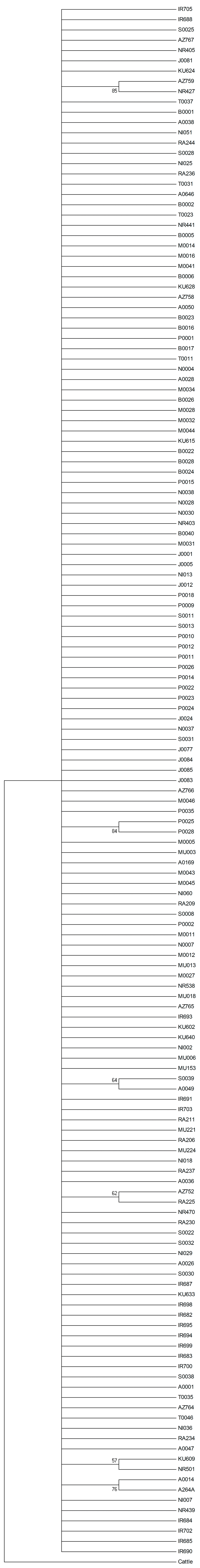

IR705
IR688
S0025
AZ767
NR405
J0081
KU624
85
AZ759
NR427
T0037
B0001
A0038
NI051
RA244
S0028
NI025
RA236
T0031
A0646
B0002
T0023
NR441
B0005
M0014
M0016
M0041
B0006
KU628
AZ758
A0050
B0023
B0016
P0001
B0017
T0011
N0004
A0028
M0034
B0026
M0028
M0032
M0044
KU615
B0022
B0028
B0024
P0015
N0038
N0028
N0030
NR403
B0040
M0031
J0001
J0005
NI013
J0012
P0018
P0009
S0011
S0013
P0010
P0012
P0011
P0026
P0014
P0022
P0023
P0024
J0024
N0037
S0031
J0077
J0084
J0085
J0083
AZ766
M0046
P0035
84
P0025
P0028
M0005
MU003
A0169
M0043
M0045
NI060
RA209
S0008
P0002
M0011
N0007
M0012
MU013
M0027
NR538
MU018
AZ765
IR693
KU602
KU640
NI002
MU006
MU153
64
S0039
A0049
IR691
IR703
RA211
MU221
RA206
MU224
NI018
RA237
A0036
62
AZ752
RA225
NR470
RA230
S0022
S0032
NI029
A0026
S0030
IR687
KU633
IR698
IR682
IR695
IR694
IR699
IR683
IR700
S0038
A0001
T0035
AZ764
T0046
NI036
RA234
A0047
57
KU609
NR501
76
A0014
A264A
NI007
NR439
IR684
IR702
IR685
IR690
Cattle
