# Supplementary figures and images for: Mitochondrial DNA Variability of Domestic River Buffalo (Bubalus bubalis) Populations: Genetic Evidence for Domestication of River Buffalo in Indian Subcontinent
Source: Genome Biol Evol. 2015 Apr 20;7(5):1252–9. doi: 10.1093/gbe/evv067 (PMC4453062; doi:10.1093/gbe/evv067)

Figure S2. Bayesian phylogenetic tree of river buffalo rooted with *Bos taurus*

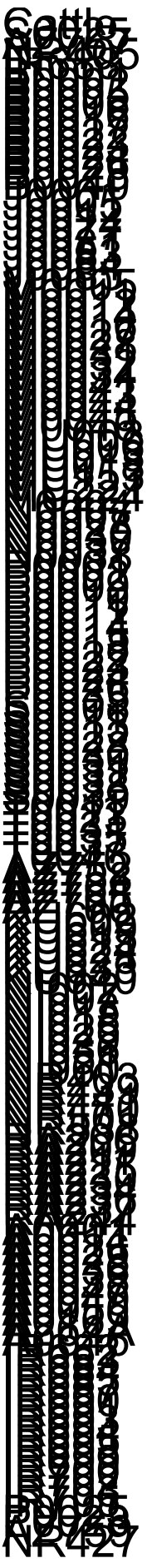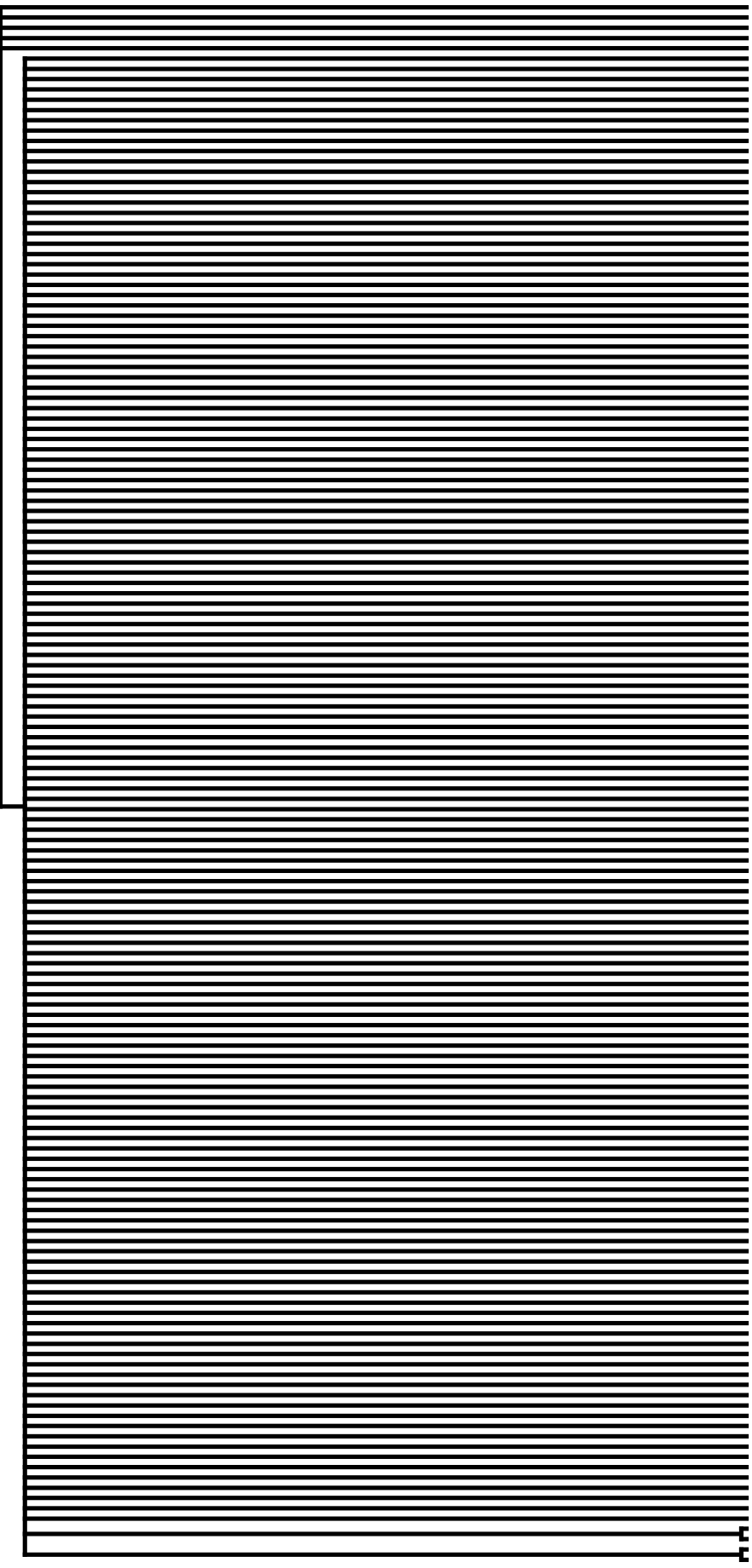

Supplement: Supplementary Data [file supp_evv067_Figure_S2.pdf]
