# Supplementary material for: Mitochondrial DNA Variability of Domestic River Buffalo (Bubalus bubalis) Populations: Genetic Evidence for Domestication of River Buffalo in Indian Subcontinent
Source: Genome Biol Evol. 2015 Apr 20;7(5):1252–9. doi: 10.1093/gbe/evv067 (PMC4453062; doi:10.1093/gbe/evv067)
Supplement: Supplementary Data [file supp_evv067_New_Microsoft_Office_Word_Document.docx]

**FIG. S1.** Maximum parsimony phylogenetic tree of river buffalo rooted with *Bos taurus*. The bootstrap values are shown above the branches.

**FIG. S2.** Bayesian phylogenetic tree of river buffalo rooted with *Bos taurus*.
