# Supplementary material for: Mitochondrial DNA Variability of Domestic River Buffalo (Bubalus bubalis) Populations: Genetic Evidence for Domestication of River Buffalo in Indian Subcontinent
Source: Genome Biol Evol. 2015 Apr 20;7(5):1252–9. doi: 10.1093/gbe/evv067 (PMC4453062; doi:10.1093/gbe/evv067)
Supplement: Supplementary Data [file supp_evv067_Supplementary_Table_S1.docx]

Supplementary Table S1. GenBank accession numbers of the buffalo sequences used in this study.

| S.No | Buffalo population | GenBank Accession number |
| --- | --- | --- |
|  | River buffalo | EF464323–EF464457  GQ166697-GQ166748  EU26883- EU26909, EU780708  DQ995682 to DQ995715 |
|  | Swamp buffalo | GQ260217-GQ260455  DQ364160-DQ364189  DQ658051- DQ658139  EF053531-EF053552  DQ995708-DQ995712  AY195596-AY195599  AF197218-AF197223  FJ873676-FJ873683  AY702618 |
